# Efficacy of cidofovir in recurrent juvenile respiratory papillomatosis

**DOI:** 10.1590/S1808-86942010000600008

**Published:** 2015-10-19

**Authors:** Fabiana Valera, Lucas Maldonato, Jessé Lima, Daniel S. Küpper, Rodrigo N. Lacerda, Rui Mamede, Hilton Ricz

**Affiliations:** 1 Post-doctoral degree, assistant professor, FMRP - USP; 2 Ex-resident of otorhinolaryngology, FMRP-USP, physician; 3 Ex-resident of otorhinolaryngology, FMRP-USP, physician; 4 Master's degree, assisting physician in otorhinolaryngology, FMRP- USP; 5 Master's degree, assisting physician in otorhinolaryngology, FMRP- USP; 6 Full professor of the Head & Neck Surgery Discipline, FMRP-USP; 7 Professor of the Head & Neck Surgery Discipline, FMRP-USP

**Keywords:** papilloma, tumor virus infections, larynx

## Abstract

The efficacy of cidofovir in juvenile recurrent respiratory papillomatosis (JRRP) remains uncertain due to the lack of published case-control studies.

**Aim:**

To establish factors affecting the progression of JRRP prognosis, and to evaluate cidofovir for eradicating JRRP.

**Study design:**

Retrospective.

**Methods:**

22 children with JRRP were evaluated at a referral center. All children underwent surgical debulking, followed by cidofovir injection (Group 2) or not (Group 1). Age at diagnosis was correlated with the Derkay score and disease outcome. Disease progression was compared between groups 1 and 2.

**Results:**

fifteen children were considered disease-free; 8 were in Group 2 and 7 in Group 1. Age and total and clinical scores (P<0.05) were negatively correlated. The mean number of surgeries required to control the disease was identical in both groups; the duration of treatment until remission was significantly higher in Group 1 (P<0,05).

**Conclusion:**

JRRP is more aggressive in earlier onset disease. The duration of treatment was significantly lower in patients treated with cidofovir until eradication of JRRP compared to patients treated with surgery only.

## INTRODUCTION

Juvenile recurrent respiratory papillomatosis (JRRP) is the most common benign tumor of the larynx and the second main cause of dysphonia in children. The incidence of JRRP has been estimated at 4.3 new cases for every 1,000 children in the United States of America.^1^ JRRP is caused by the human papilloma virus (HPV); the subtypes 6 and 11 have been reported as being most frequently associated with recurrent respiratory papillomatosis^1^, ^2^, ^3^, ^4^ in the pediatric population. The main form of transmission in children is vertical, during pregnancy.^1^ Recurrent respiratory papillomatosis affects mostly primiparous children of young mothers in normal delivery.^1^, ^2^, ^4^

JRRP is a benign disease, but has an unfavorable prognosis because of frequent relapses and dissemination across the aerodigestive tract; there is also the risk of malignant degeneration.^4^ There is a 3.6 higher chance of requiring over four surgeries per year, and a nearly twofold chance of the disease involving more than one anatomical site, when the diagnosis is made before age 3 years.^5^ Other poor prognostic factors of JRRP are subglottic extension and tracheostomy.^6^

The current therapy for JRRP is surgery, either using laser or the microdebrider. As no approach can eradicate JRRP, the main goal of surgery is to remove lesions at the same time preserving adjacent anatomical structures^1^ to avoid synechiae or stenoses. However, papillomas recur rapidly in some patients and require more than four surgeries per year; or they may present disease in multiple sites.^1^ In these cases, adjuvant therapy is indicated in an attempt to improve the outcome.

Intralesional injection of cidofovir (Vistide®, Gilead, Foster City, CA, US) has been encouraged by several case series of recurring JRRP, although the dose has not been standardized. Cidofovir is the most frequently used adjuvant treatment for this condition in the US;^1^ according to reviews by the American Society of Pediatric Otorhinolaryngology (ASPO) and the British Association of Pediatric Otorhinolaryngology (BAPO), about 10% of JRRP patients are given adjuvant therapy with cidofovir.^7^

The purpose of this study was to evaluate the effect of age on the diagnosis and the extent of disease on the progression of JRRP, and to assess what effect cidofovir has on eradication of JRRP. Children treated successfully with surgery alone were compared with children treated successfully with surgery and intralesional injection of cidofovir.

## MATERIALS AND METHODS

Before 2006, two disciplines carried out identical laryngeal surgeries at our institution, namely the otorhinolaryngology and the head & neck surgery clinics; they are currently joining efforts and reviewing their approach of laryngeal surgery.

The otorhinolaryngology team proposed cidofovir as a treatment option after the hospital approved the purchase of this drug for the treatment of JRRP in 2003. At present, all children with JRRP treated at this hospital are given adjuvant cidofovir therapy. Because of cost, this drug has been approved only for the otorhinolaryngology unit; the head & neck surgery unit offers only surgery. This bias has generated two groups treating JRRP differently; one group systematically is treated with cidofovir, and the other is not, therefore comprising a control group for this study.

Thus, we carried out a retrospective study of 22 pediatric patients undergoing removal of laryngeal papillomas from 2000 to 2008. Only patients in whom symptoms and lesions regressed fully for at least 6 months as evaluated on laryngeal endoscopy. Patients lost to follow-up, or where records were insufficient for this study, or patients with residual disease were excluded. Children in both groups underwent laryngeal surgery under general anesthesia for removal of papillomas; apparently tumor-free neighboring structures were preserved. In the adjuvant therapy group, cidofovir was injected after surgical hemostasis.

The Derkay^1^ scale was applied in each procedure to classify the lesions and the severity of JRRP. Group 1 patients (control) underwent further surgeries when symptoms manifested; group 2 children (treated with cidofovir) systematically underwent a second surgery one month after the first procedure to reinject the drug, even if there are no signs of recurrences. Next, these children were reassessed every 15 to 30 days; surgery was done if there were any signs of recurrences, with or without symptoms. As cidofovir was injected at longer intervals than those reported in the literature, a 15 mg/mL concentration was used, within the maximum dose of 1 mg/kg of weight. Only two surgeons performed all cidofovir injections. Renal and hepatic function, coagulation and a complete blood count were evaluated periodically; thus far, no disorders have been documented.

The age at diagnosis of JRRP was correlated with: 1) disease aggressiveness according to the Derkay^1^ clinical, anatomical and total scales; 2) tendency to recur in relation to the moment of treatment and the number of surgeries required for controlling the lesions. Spearman's correlation test was applied to calculate these correlations, which were defined as significant when p<0.05.

The progression of both treatment groups was compared in terms of the total number of surgeries per patient and the duration of treatment. The mean interval between surgeries and Derkay's mean total score in each surgery (except for the diagnostic procedure) were compared for a better description of groups. The Mann-Whitney non-parametric test was applied to analyze these parameters; the significance level was p<0.05.

The hospital institutional review board approved this study (process no. 11227/2008).

## RESULTS

The sample consisted of 22 patients, 12 female and 10 male. The age at diagnosis ranged from 10 to 99 months (mean: 45.59 ± 23.96). The initial clinical score ranged from 1 to 11 points (mean: 5.18 ± 3.36). Dysphonia was the most common symptom; it was present in all 22 patients, and was moderately intense in 17 patients. Stridor was present in 10 cases, 5 with at least moderate intensity.

The most commonly affected site are the vocal folds (20 of 22 cases; bilateral in 15 cases), followed by the vestibular folds (15 cases, bilateral in 10 cases). The anterior commissure (11 cases) and the subglottis (8 cases) were also affected, albeit less frequently. The mean anatomical score was 10.5 ± 5.11, and the mean total score was 15.68 ± 7.08.

**Figure 1:**
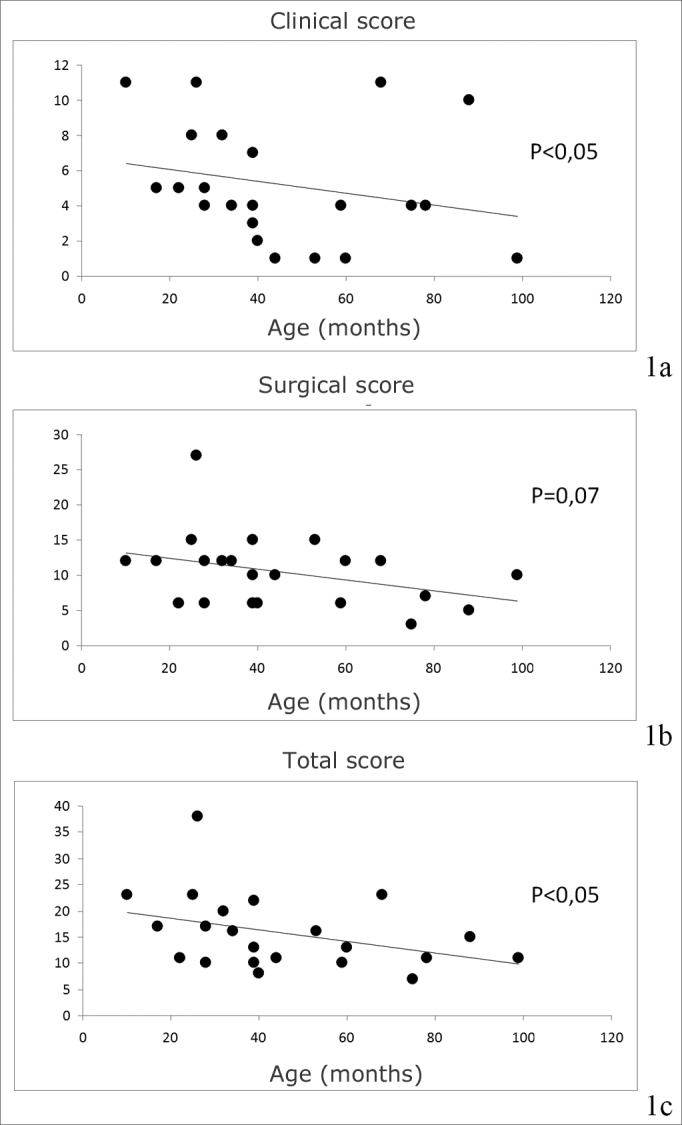
Correlation between age at diagnosis and the Derkay score: 1a: clinical score; 1b: anatomical score; 1c: total score.

These scores were correlated with age of diagnosis; there was a significant negative correlation between age and the clinical/total scores (r = -0.4413 / r = -0.4349; p<0.05 for both). Age of diagnosis and the anatomical score (r = -0.3922; p=0.07) trended towards a negative correlation (Fig. 1). However, there was no correlation between age at diagnosis and the number of surgeries or the required duration of treatment for disease remission.

Before the comparison between treatment groups, 7 patients were excluded; 4 were lost to follow-up and 3 still had active lesions upon evaluation. There were 15 patients in this phase of the study, 7 in group 1 (surgical treatment only) and 8 in group 2 (surgical treatment following by cidofovir injection). Table 1 shows the data on each group.

**Table 1:** Comparison of treated groups; group 1 was the control and group 2 consisted of patients treated with cidofovir. Statistical comparison based on the Mann-Whitney no-parametric test.

	Group 1		Group 2		
	Mean	SD	Mean	SD	P
Number of surgeries	6.71	4.71	6.50	3.96	> 0.9999
Duration of treatment	50.71	35.16	20.00	15.97	0.0440
Interval between surgeries	11.62	10.96	3.42	1.65	0.0289
Mean total score	13.22	3.75	5.42	2.30	0.0006

The mean number of surgeries needed to control the disease was similar in both groups (6.71 ± 4.71 in group 1 and 6.50 ± 3.96 in group 2, p>0.05). But the duration of treatment until disease remission was significantly higher in group 1 (50.71 ± 35.16 months) compared to group 2 (20 ± 15.97 months) (p<0.05). This difference occurred because the interval between surgeries was significantly shorter in group 2 (3.42 ± 1.65 months) compared to group 1 (11.62 ± 10.96 months) (p<0.05), as was the mean total score during surgeries for recurrence (5.42 ± 2.30 in group 2 *vs.* 13.22 ± 3.75 in group 1; p<0.001).

## DISCUSSION

JRRP is generally diagnosed before the age 5 years; the age distribution at this point is equal.^4^ Our results concur with other published data. The mean age at diagnosis was 45 months and the gender distribution was similar.

The main site of papillomas was the vocal folds, followed by the vestibular folds and subglottic extension. Thus, dysphonia was the most common symptom, followed by stridor. Five of these children required tracheostomy during treatment; 3 were in the cidofovir group and 2 were in the control group. Decannulation was successful after disease control in all cases.

No papillomatous extension to the trachea or beyond the respiratory tract was seen in our study samples, probably because of the inclusion criteria for this retrospective study; only children with disease remission for at least 6 months (considered successfully treated cases) were included. Although this criterion was important for comparing treatment protocols, it obviously resulted in a bias by excluding children with more extensive disease (and therefore a worse outcome).

JRRP is more aggressive - more respiratory symptoms and disease extension - the lower the patient's age. This concurs with other published results.^1^ However, more extensive disease was not necessarily related with increased difficulty in disease remission; no significant correlation was found between age and disease extension and the duration of treatment.

The surgical technique was similar in both groups; micro-scissors or cutting forceps only were used. Laser or microdebriders were not used in any of these patients. There was a difference in the indication of surgery if the disease recurred: surgery was indicated for any sized tumor in the cidofovir group, whereas surgery was done in the control group only if there were symptoms (dysphonia or breathing difficulty). Thus, more surgeries at shorter intervals and more limited lesions after each procedure were expected in the cidofovir group. In fact, the mean interval between surgeries and the Derkay mean score during these procedures were significantly lower in the cidofovir-treated group. Nevertheless, the mean number of procedures required to control JRRP was similar in both group. The treatment time for eradication of JRRP was significantly lower in the cidofovir-treated group compared to controls.

Our results suggest that cidofovir-treated children have a better prognosis compared to those treated surgically only; the duration of treatment until eradication of JRRP was shorter in the former. These results concur with recently published studies,^1^, ^3^, ^4^, ^5^ in particular with Chadha et al.'s^7^ review, which suggested that cidofovir may in fact be an adjuvant treatment for JRRP.

At this point only one double-blind randomized study of cidofovir for the treatment of JRRP has been published;^8^ however, if included adult and pediatric patients. Furthermore, because of FDC requirements, the initial dose in that study was 0.75 mg/mL in adults and 0.3 mg/mL in children, which was rather different than the routinely recommended doses in the literature. Under such conditions, the authors found no differences between the cidofovir-treated group and controls.

The study protocol differs from others in the literature in that, for logistical issues, we were unable to carry out surgeries every two weeks in all patients in the cidofovir protocol. This situation required us to carry out periodical assessments, and surgeries were indicated based on the results of laryngeal endoscopy. Thus, the study group was more similar to the control group, and a truer comparison of certain variables that would not have been evaluated in more usual protocols in published studies became possible.

On the whole it was not possible to state that the superior results of group 2 were due to the use of cidofovir, since this group was not matched to the control group in relation to treatment interval or indication of surgery for recurrences. The study was also not randomized or double-blind. However, ours results suggest that in children with JRRP who progressed favorably, cidofovir was an import adjuvant co-factor to the traditional surgery; it decreased the treatment time for disease remission.

## CONCLUSION

We may conclude that:

- JRRP is more aggressive (on symptoms and diseases extension) the younger the child at the moment of diagnosis.
- Patients treated with cidofovir had a significantly lower duration of treatment until eradication of JRRP compared to the group treated surgically only.
